# Gastroesophageal Reflux and Body Movement in Infants: Investigations with Combined Impedance-pH and Synchronized Video Recording

**DOI:** 10.1155/2011/271404

**Published:** 2011-05-24

**Authors:** Tobias G. Wenzl, Olaf Stoltenburg, Jiri Silny, Heino Skopnik

**Affiliations:** ^1^Klinik für Kinder-und Jugendmedizin, Universitätsklinikum der RWTH Aachen, Pauwelsstraße 30, 52074 Aachen, Germany; ^2^Helmholtz Institut für Biomedizinische Technik, RWTH Aachen, Pauwelsstraße 20, 52074 Aachen, Germany

## Abstract

The aim of this paper was to investigate the temporal association of gastroesophageal reflux (GER) and body movement in infants. GER were registered by combined impedance-pH, documentation of body movement was done by video. Videorecording time (Vt) was divided into “resting time” and “movement time” and analyzed for occurrence of GER. Association was defined as movement 1 minute before/after the beginning of a GER. Statistical evaluation was by Fisher's exact test. In 15 infants, 341 GER were documented during Vt (86 hours). 336 GER (99%) were associated with movement, only 5 episodes (1%) occured during resting time. Movement was significantly associated with the occurrence of GER (*P* < .0001). There is a strong temporal association between GER and body movement in infants. However, a clear distinction between cause and effect could not be made with the chosen study design. Combined impedance-pH has proven to be the ideal technique for this approach.

## 1. Introduction

The role of gastroesophageal reflux (GER) and its association with various extraesophageal symptoms have been studied previously in children [1]. The combined measurement of multiple intraluminal impedance and pH (MII-pH) is increasingly accepted as the primary diagnostic tool in this situation [2]. Irritability and dystonic body movements in infants with regurgitation are a common clinical observation [3–5]. The aim of this study was to investigate the temporal association of body movement and GER in infants, by combining MII-pH [6, 7] with continuous, synchronized video recording.

## 2. Patients and Methods

Fifteen infants (mean age at study 97 ± 52 days; 8 female, 7 male) with recurrent regurgitation were examined over approximately 6 hours each. None received medication to control GER. They were fed their regular milk formula and kept supine after feeding.

The combined technique of MII-pH has been described in detail previously [6, 8]. In this study, a standard flexible catheter (outer diameter 1.5 mm) with a pH-sensitive antimony electrode and six impedance channels (Helmholtz Institut, Aachen, Germany) was placed in the esophagus under fluoroscopy. Distance between impedance channels was 1.5 cm, over a total measuring length of 9 cm. Measuring segments were positioned from just above the cardia (channel 6) to the hypopharynx (channel 1), with the pH sensor situated at the level of channel 5, that is, approximately 3 cm above the gastroesophageal junction. The acquisition rate of pH and impedance signals was 50 Hz per channel. For analysis, MII-pH data was stored on a standard recording device with implemented custom software (MOT 2.01, Helmholtz Institut, Aachen, Germany) [9].

For movement documentation, video recording was performed with a standard video camera (PC224, Panasonic, Hamburg, Germany) positioned at the left side of the bed. The video signal was recorded simultaneously and picture-in-picture together with the MII-pH signal with a recording system at the bedside.

MII-pH data were visually analyzed for the typical impedance pattern of GER, indicated by a retrograde esophageal volume flow [6]. In this study, GER was diagnosed only if this typical pattern was noted in the esophageal impedance. Documentation during each GER included the minimal pH value, the maximal height reached by the refluxate, and the GER duration, defined as time needed to reach 50% of the initial impedance value in the most distal impedance channel [9].

The total analyzable time with video and MII-pH recording in all patients was defined as “video time” (Vt). GER episodes that occured during Vt were further analysed.

All video recordings were analysed, and all body movements during Vt were documented. Per definition, phases with visible movement of the infant during Vt were called “movement time” (MT), phases without visible movement during Vt were called “resting time” (RT). To discriminate between RT and MT during prolonged periods of movement, the duration of a resting episode had, by arbitrary definition, to be ≥10 sec to be registered as such, that is, episodes of movement that were interrupted by a resting phase <10 sec were considered as one movement episode. For further consistent statistical analysis, MT and RT were divided into intervals of 120-second duration.

The occurrence of a body movement episode during 60 secs preceding or following the beginning of a GER (GER window time, GWT) was defined as temporal association. Accordingly, the remaining Vt was dvided into 120-second intervals (non-GER window time, nGWT) [10].

Statistical analysis was performed using Fisher's exact test. Significance was established by a *P* value <.05.

The study protocol was approved by the Ethics Committee of the Medical Faculty, RWTH Aachen, Germany. Before commencing any evaluation of an infant, informed consent was obtained from the parents.

## 3. Results

During a total measurement time of 115 h 37 min, 462 episodes of GER were registered in the 15 infants.

341 of these GER occured during Vt (86 h 46 min) and formed the database for the following analysis.

Fifty-eight (17%) GER episodes were acidic (pH < 4). Most GER episode (212 of 341; 62.2%) reached the hypopharynx (MII channel 1). In 29.6%, the maximal height reached by the refluxate was channel 2, in 5.0% channel 3, and in 3.2% channel 4, respectively. Total GER duration in all patients during Vt was 1 h 36 : 50 min (mean 17 sec ± 18 sec).

Total resting time (RT) in all patients during analyzable video time (Vt) was 62 h 8 min (mean 4 h 8 min ± 1 h 15 min), resembling 72% of Vt. Total movement time (MT) in all patients during Vt was 24 h 38 min (mean 1 h 38 min ± 37 min), resembling 28% of Vt.

336 (98.5%) of the 341 GER were associated with body movement within the two-minute interval around the beginning of each GER episode (GER window time, GWT). In 5 GER (1.5%), no movement was documented during GWT.

**Figure 1:**
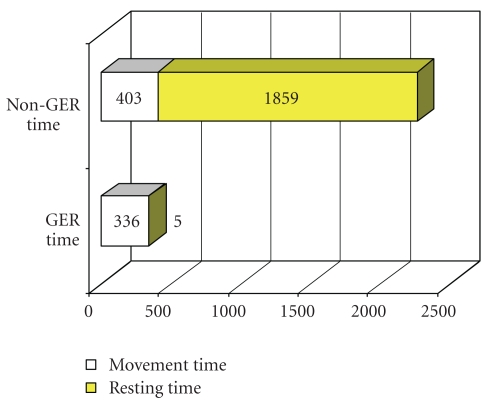
Association of GER with movement time and resting time. Non-GER time = 2 minute-intervals without GER, GER time = 2 minute intervals before/after GER, Movement time = 2 minute intervals with body movement, and Resting time = 2 minute intervals without body movement.

The remaining non GER window time (nGWT) of 75 h 24 min was subdivided according to the documented movement of the infants into 403 (17.8%) two-minute intervals of movement time (MT) and 1859 (82.2%) two-minute intervals of resting time (RT) (Figure 1).

When compared to GER-free measuring time, movement time was significantly associated with the occurrence of GER (*P* < .0001).

## 4. Discussion

An association with GER is presumed for various extraesophageal symptoms [1]. The results described here demonstrate an association of movement episodes and gastroesophageal reflux in infants. During 336 of 341 (98.5%) two-minute intervals surrounding a GER episode, a body movement of the infant was documented, only 5 of 341 (1.5%) two-minute intervals surrounding a GER episode were without movement. This result was highly significant when compared to the GER-free measurement phases, where body movement of the infants was documented in less than 20% of the measurement time.

Gastroesophageal reflux is frequent in infants and may constitute a physiological phenomenon that triggers various extraesophageal reactions [3, 4]. The clinical observation, that body movements in infants occur more frequently in the phases around GER events [11], could be confirmed with this study. Whether these movements are a sign of arousal prior to a GER episode or means of, for example, facilitating GER clearance by altering the position and slope of the esophagus for protection against potentially damaging GER content, could not be clarified with the chosen study design. In line with the current guideline, the exclusive use of pH monitoring is not suitable for the detection of symptom-associated GER in infancy [2, 12], the combined multiple esophageal impedance-pH recording, which has now finally made its way from bench to bedside in all age groups, is clearly superior [2, 10]. Further studies are needed to investigate the relation of cause and effect.

##  Conflict of Interests

T. G. Wenzl received research support from AstraZeneca, he received research support and served as a consultant for Sandhill Scientific and received research support and served as a speaker for Tecnomatix Germany; he also served as a speaker for Reckitt Benckiser and received research support from Johnson & Johnson. The other authors declare no conflict of interest. No honorarium, grant, or other form of payment was received for producing the paper.

## References

[1] Shields MD, Bateman N, McCallion WA, van Wijk MP, Wenzl TG (2011). Extra-oesophageal reflux disease in children. *Alimentary Pharmacology & Therapeutics*.

[2] Vandenplas Y, Rudolph CD, Di Lorenzo C (2009). Pediatric gastroesophageal reflux clinical practice guidelines: joint recommendations of the North American Society for Pediatric Gastroenterology, Hepatology, and Nutrition (NASPGHAN) and the European Society for Pediatric Gastroenterology, Hepatology, and Nutrition (ESPGHAN). *Journal of Pediatric Gastroenterology and Nutrition*.

[3] Ariagno RL, Guilleminault C, Baldwin R, Owen-Boeddiker M (1982). Movement and gastroesophageal reflux in awake term infants with “near miss” SIDS, unrelated to apnea. *Journal of Pediatrics*.

[4] Jeffery HE, Heacock HJ (1991). Impact of sleep and movement on gastro-oesophageal reflux in healthy, newborn infants. *Archives of Disease in Childhood*.

[5] Feranchak AP, Orenstein SR, Cohn JF (1994). Behaviors associated with onset of gastroesophageal reflux episodes in infants: prospective study using split-screen video and pH probe. *Clinical Pediatrics*.

[6] Wenzl TG, Moroder C, Trachterna M (2002). Esophageal pH monitoring and impedance measurement: a comparison of two diagnostic tests for gastroesophageal reflux. *Journal of Pediatric Gastroenterology and Nutrition*.

[7] Mousa HM, Rosen R, Woodley FW (2011). Esophageal impedance monitoring for gastroesophageal reflux. *Journal of Pediatric Gastroenterology and Nutrition*.

[8] van Wijk MP, Benninga MA, Omari TI (2009). Role of the multichannel intraluminal impedance technique in infants and children. *Journal of Pediatric Gastroenterology and Nutrition*.

[9] Skopnik H, Silny J, Heiber O, Schulz J, Rau G, Heimann G (1996). Gastroesophageal reflux in infants: evaluation of a new intraluminal impedance technique. *Journal of Pediatric Gastroenterology and Nutrition*.

[10] Pilic D, Fröhlich T, Nöh F (2011). Detection of gastroesophageal reflux in children using combined multichannel intraluminal impedance and pH measurement: data from the German Pediatric Impedance Group. *Journal of Pediatrics*.

[11] Vandenplas Y, Hauser B (2000). Gastro-oesophageal reflux, sleep pattern, apparent life threatening event and sudden infant death. The point of view of a gastro-enterologist. *European Journal of Pediatrics*.

[12] Salvatore S, Arrigo S, Luini C, Vandenplas Y (2010). Esophageal impedance in children: symptom-based results. *Journal of Pediatrics*.

